# Successful establishment and five-year sustainability of a neonatal-specific antimicrobial stewardship program in a low middle-income country

**DOI:** 10.3389/fphar.2022.1076392

**Published:** 2023-01-04

**Authors:** Faouzi I. Maalouf, Therese Saad, Ramia Zakhour, Khalid Yunis

**Affiliations:** ^1^ Division of Neonatology, Department of Pediatrics and Adolescent Medicine, American University of Beirut Medical Center, Beirut, Lebanon; ^2^ Department of Pharmacy, American University of Beirut Medical Center, Beirut, Lebanon; ^3^ Division of Pediatric Infectious Diseases, Department of Pediatrics and Adolescent Medicine, American University of Beirut Medical Center, Beirut, Lebanon

**Keywords:** neonates, antimicrobial stewardship, prematurity, antibiotic usage rate, quality improvement

## Abstract

**Introduction:** Antibiotic use varies substantially among neonatal intensive care units (NICUs) without any appreciable impact on outcomes. An increased use of antimicrobials has been reported in low-middle income countries. This raises the concern for potential overuse of antibiotics in a fragile patient population, thus increasing the rates of multidrug resistant organisms and affecting the developing microbiome. The presence of a neonatal-specific antimicrobial stewardship program can aid with the judicious use of antibiotics in the neonatal population and thus decrease the overuse of such medications.

**Methods:** In this quality improvement project, we established and implemented a neonatal-specific antimicrobial stewardship program with the aim of reducing antimicrobial use in the neonatal intensive care units within a year of starting. Several interventions using a multidisciplinary approach included implementing standard algorithms, direct audit and feedback, and automated hard stops.

**Results:** These series of interventions led to a 35% decrease in antimicrobial usage in the first 3 months and further decrease was seen with a median of 63% decline for a total of 5 years after project implementation. The use of the most commonly prescribed antibiotics, ampicillin and gentamicin, decreased by 63% and 79%, respectively. There was no evidence that this change in practice affected or jeopardized patient outcomes. Additionally, it showed sustainability and resilience despite the many challenges such as COVID-19 pandemic, political and financial unrest, and healthcare sector collapse.

**Discussion:** This model-based and multidisciplinary low-cost approach can lead to marked improvement affecting neonatal outcomes and can be replicated in other similar centers.

## Introduction

Despite the major advances in neonatology during the last decades, neonatal sepsis, defined as the systemic manifestation of infection during the first 28 days of life, is still a main cause of admission to the neonatal intensive care unit (NICU) leading to neonatal morbidity and mortality (Klingenberg et al., 2018). Among all medications prescribed in the NICU, antibiotics are the most used, a trend that has persisted with time (Clark et al., 2006; Hsieh et al., 2014).

In a recent global point-prevalence study, 26% of infants admitted to the NICU were receiving antibiotics on a given day, with higher proportions reported in low-to-middle income countries compared to high-income countries LMIC. Antimicrobial use varies substantially among NICUs, with usage rates ranging from 2.4% to 97.1% of patient days (Schulman et al., 2015). Such variation in usage rates comes without any appreciable impact on outcomes. A global report showed that units with an antimicrobial stewardship program ASP have lower antibiotic usage (Prusakov et al., 2021).

Antimicrobial use in neonates, particularly those who are ill and premature, can result in various direct adverse effects such as nephrotoxicity and hematological abnormalities. Moreover, the misuse of antimicrobials, especially the overuse of broad-spectrum antibiotics for premature infants, has been shown to be associated with increased risk of death and necrotizing enterocolitis (NEC) as well as increased rates of invasive candidiasis (Cotten et al., 2006; Cotten et al., 2009; Cantey et al., 2018). Finally, use of broad-spectrum antibiotics can lead to the emergence of multi-drug resistant organisms (MDROs), one of the major public health challenges of the century (van de Sande-Bruinsma et al., 2008). In the long term, the disruption that antimicrobial use in preterm infants causes to gut microbiome might increase the risk of chronic metabolic and immune disorders and affect their growth (Gasparrini et al., 2019; Uzan-Yulzari et al., 2021).

In an attempt to reduce healthcare costs and improve quality of care, the American Board of Internal Medicine Foundation launched the “Choosing Wisely” campaign in 2011. Translating this campaign to newborn medicine, one of the first interventions identified as beneficial was avoiding the routine use of antibiotics beyond 48 h for asymptomatic neonates (Ho et al., 2015). Since then, antimicrobial stewardship has been highly endorsed by the american academy of pediatrics and neonatologists have been among the pioneers in stewardship in the pediatric world. The Infectious Diseases Society of America has set core elements that help establish antimicrobial stewardship programs and implement initiatives that standardize antimicrobial use. (Dellit et al., 2007).

Several guidelines address the management of neonates with sepsis and attempt to standardize the initiation of antibiotic therapy in term neonates. Yet, at the time of study initiation, none clearly delineated the choice nor the duration of therapy for both term and preterm infants. This lack of clear guidance led to variation of care. Recently, the American Academy of Pediatrics updated its guidelines on the management of suspected neonatal sepsis for both term and preterm neonates, a step that could consolidate the care for this population (Puopolo et al., 2018a; Puopolo et al., 2018b).

In Lebanon, a (LMIC), approximately one in ten babies is born prematurely and might need admission to the NICU (Chawanpaiboon et al., 2019)**.** To date, there are no published data on the incidence of neonatal sepsis and the antimicrobial usage rates in NICUs of Lebanese hospitals. However, data from the Middle Eastern region shows a concerning incidence of up to 60% resistance to ampicillin and gentamicin in bacteria isolated from neonates with sepsis (Khalil et al., 2020). Similar trends in antimicrobial resistance in other LMICs, such as India, Nigeria, the Democratic Republic of the Congo, Pakistan, and China, have been reported, where MDROs are responsible for 214,000 neonatal deaths each year, (Laxminarayan et al., 2016).

Until the time of this study, there had been no official neonatal-specific antimicrobial stewardship program (NS-ASP) established in any Lebanese hospital. In this quality improvement project, we report a NS-ASP aiming at guiding prescription and decreasing the unnecessary use of antibiotics in the NICU. Our goal was to decrease antibiotic usage by 20% in the first 6 months of implementation and maintain the downward trend for at least 1 year thereafter. We also share the approach in a resource-limited setting to implement and maintain such a program over a period of 5 years.

## Materials and methods

### Setting

This is a prospective, single-center quality improvement project conducted at the (NICU) at the American University of Beirut Medical Center in Beirut, Lebanon. Our NICU is a 23-bed level IV closed unit with approximately 250 admissions per year. It is a major referral center with 30% of patients being out born. We defined neonatal sepsis as early onset (EOS) if it occurred ≤72 h of life and late onset if it occurred >72 h of life.

The project included a retrospective chart review phase followed by a prospective implementation phase with multiple Plan-Do-Study-Act (PDSA) cycles. The retrospective chart review included patients admitted to the NICU at any time between 1 January 2015, and 31 December 2015.

This project was exempt from Institutional Review Board approval and was approved by the hospital Quality Advisory Council.

#### Retrospective chart review phase (January 2015—December 2015)

To understand the baseline usage of antibiotics in our NICU, a retrospective review of charts of all neonates who received antibiotics from 01 January 2015, till 31 December 2015, was performed. The duration of 1 year was chosen to account for seasonal variation and differences in practice among the three neonatologists at that time. The range of data was 2 years prior to the project initiation as the charts were scanned and stored on electronic servers at that time making this time frame available for review for this study.

Patients were included if they were admitted to the NICU and received intravenous or intramuscular antimicrobials including antibacterials and antifungals during their hospitalization. Patients were excluded if they were transferred from other hospitals while being treated with antibiotics. Data on antimicrobials, including dosage, start and end dates, and dosing intervals were collected. Antibiotic usage rate (AUR) was calculated as the days of therapy (DOT) divided by 1,000 patient days (CDC Library (U.S.), 2022). AUR was plotted for each month and then compared to the rates after project implementation.

Chart review was validated by having two independent reviewers collecting the baseline data separately and comparing findings. Data collected also included gestational age, sex, age at antibiotic initiation, mode of delivery, maternal risk factors (including fever, rupture of membranes, GBS status, and prenatal antibiotics), signs of sepsis, antibiotic(s) used, duration of use, documented indication/justification of use, and laboratory workup including blood, urine, and cerebrospinal fluid.

## Interventions

The implementation phase took place between 01 April 2017 and 31 March 2022.

### PDSA 1 (April 2017—August 2017)

A multidisciplinary team was formed including two neonatologists, a pediatric infectious disease specialist, a pediatric clinical pharmacist, and a neonatal nurse. The role of the team was to implement a NS-ASP and monitor antibiotic usage at different intervals. Educational sessions to faculty and staff on the benefit of NS-ASP and harm of antibiotic overuse were also delivered at the beginning of the project. Topics included in the sessions were etiologies of neonatal sepsis based on newborn’s age, the adverse effects of antimicrobial overuse, and the increased incidence of multi-drug resistance.

The NS-ASP team performed biweekly audit and feedback rounds on patients taking antibiotics, and a neonatal clinical pharmacist rounded daily with the clinical team and inquired about the need and duration of antimicrobial therapy. During the “ASP round,” the charts of patients on antibiotics were reviewed specifically looking at the reason for prescription, choice of antibiotic, and expected duration of treatment. The NS-ASP team would give a recommendation to the clinical team and left the final decision to the attending physician.

### PDSA 2 (September 2017—October 2018)

**FIGURE 1:**
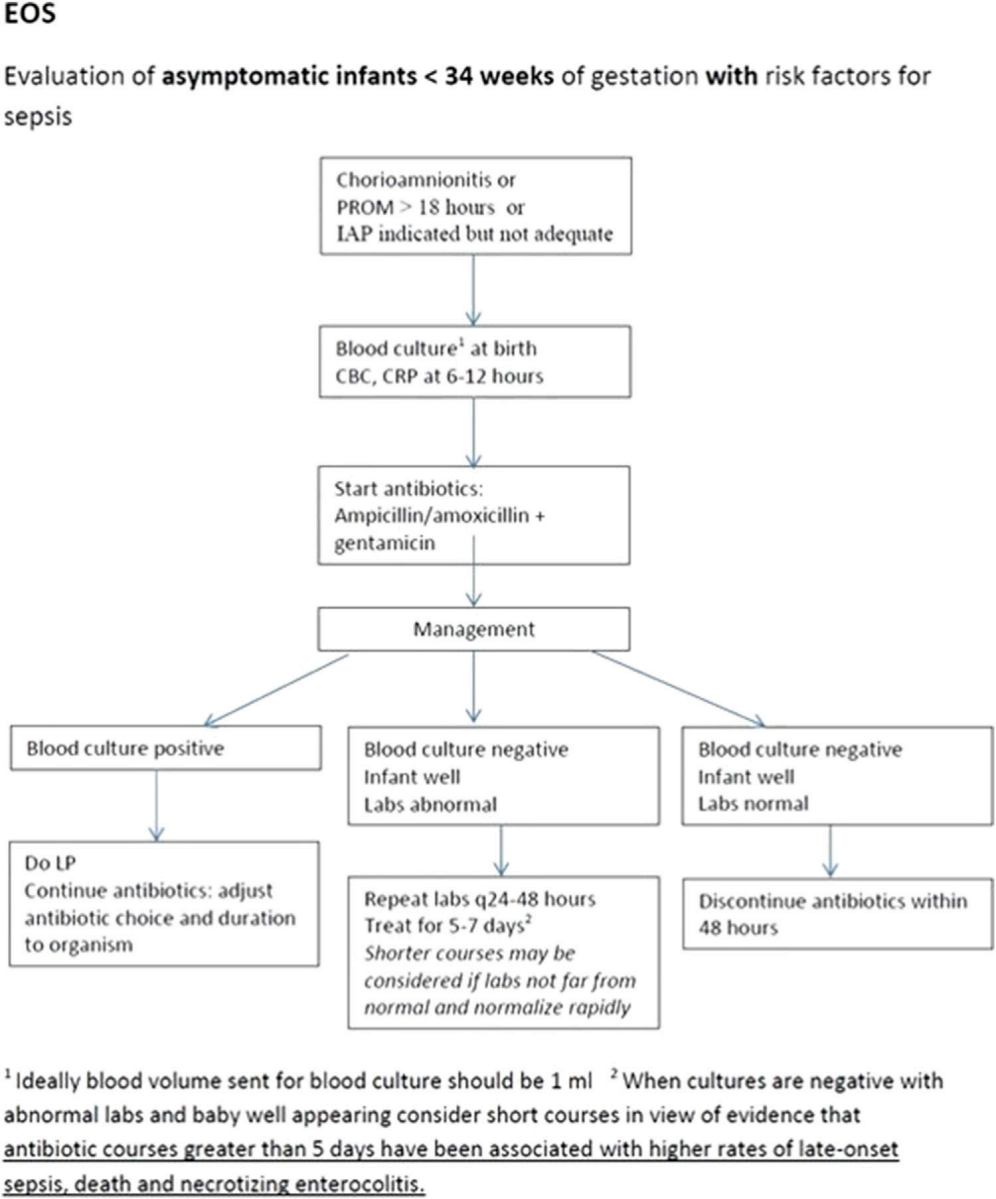
An example of the algorithm used to guide the work up of neonates with suspected sepsis.

The NS-ASP team met on a monthly basis, during which they reviewed the data collected and discussed further interventions. Variation in antimicrobial prescribing practices was noted among the different neonatologists. To address this, the next intervention was to establish different algorithms to guide treatment based on different guidelines including the American Academy of Pediatrics and World Health Organization recommendations, also factoring in local sensitivity patterns, drug availability, and expert opinion. An example of one of the unit algorithms can be seen in Figure 1. These algorithms were used during regular ASP rounds in order to approach the clinical team. Manual data collection persisted during that phase.

### PDSA 3 (November 2018—February 2020)

During this period, the medical center had transitioned into a complete electronic health record (EHR) system. Resources during that time were allocated towards EHR implementation which affected the ongoing workflow of the NS-ASP team. However, ASP work continued, and the new intervention was to include EOS algorithms into electronic order sets and create an automated NS-ASP report that could be compiled on different intervals which enabled easier access to data in a prospective manner in addition to more accurate data reporting *via* pharmacy dispensing and drug administration data.

### PDSA 4 (March 2020—April 2022)

With the decrease in AUR noted in real-time, the team focused on new interventions that would solidify the new practices in addition to utilize the strengths of the EHR. A 3-year retrospective review of laboratory data investigated time to positivity of blood cultures and the mean duration for cultures to grow an organism was 22.9 h. A safe decision was taken to implement a hard-stop at 48 h for antibiotics prescribed for suspected EOS. This was embedded in the electronic order set with an option to opt out if needed by the prescribing clinician. Educational sessions were delivered to the NICU team informing them of the new order set and criteria to use. A daily Best Practice Alert was sent to the prescribing team as a safety net. Usage of the 48-h automatic stop was tracked by the ASP team.

### Measures

#### Outcome meazsure

The primary outcome measure was AUR, accounting for the DOT for all antimicrobials per 1,000 patient-days. DOT was defined as the number of days of giving an IV or IM antibiotic which was calculated from the start and end dates of prescription. This was obtained from the hospital pharmacy dispensing data, which initially was a manual task then was upgraded to an automated report. Medication administration data was also used to identify patients receiving antibiotics through an automatically generated report from the HER system. The secondary outcome was the mean length of stay (LOS) defined as the duration a patient stayed in the NICU from day of admission to the day of discharge.

#### Process measure

Compliance to the use of the 48-h automatic stop order was defined as a process measure. This parameter was measured *via* an automated report by the EHR. Compliance was reported as the percentage of occurrences the team used the order sets containing the automatic stop out of the total times antibiotics were prescribed for EOS.

#### Balancing measures

To monitor the safety of the interventions and make sure no harm was attributed to the decrease in antibiotic use, several balancing measures were tracked including mortality rate, which was defined as number of deaths in the NICU divided by 100 admissions, and number of positive blood cultures. Certain organisms that grew after 24 h of incubation were considered contaminants after review by the NS-ASP team and hospital infection control.

### Analysis

A run chart was plotted to evaluate the trend of AUR, and this was updated monthly. Demographics were expressed as means, percentage and frequencies. For parametric data, Fisher exact test was used, and for continuous variables *t*-test was used. LOS was compared at baseline and after project implementation. Statistical differences in LOS were analyzed using ANOVA. A *p*-value of less than .05 was considered statistically significant. The data was analyzed using IBM SPSS Statistics (Version 28).

## Results

A total of 716 charts were reviewed in this study. The retrospective baseline data included 153 patients whereas the prospective intervention period of 5 years included 563 patients. Baseline and post-intervention patient demographic characteristics were similar in terms of gestational age, sex, and birth weight (see table 1). There were two statistically significant differences seen between the patients in the different study groups. The proportion of patients treated for EOS decreased from 77% to 39% (*p* < .001) and the age at antibiotic initiation increased from 4.1 to 13.9 days (*p* < .001).

**TABLE 1:** Patient characteristics during the two periods of the study.

Characteristic	Pre-intervention, N = 153	Post-intervention, N = 532	p-value
Gestational age, in weeks (mean ± SD)	34 ± 0.4	33.8 ± 2.7	.784
Birth weight, in grams (mean ± SD)	2462 ± 149	2234 ± 543	.23
Sex, %			
Male	58.2	57	.446
Female	41.8	43	
Treatment for early onset sepsis, %	77	39	<.001
Day of life on antibiotic initiation, day (mean ± SD)	4.1 ± 1.6	13.9 ± 16.9	<.001
Length of stay, days (mean ±SD)	23.6 ± 5.5	41.6 ± 31.8	.062
Antibiotic usage rate, per 1,000 patient days (median, IQR)	767.1	434.8	<.001

**FIGURE 2:**
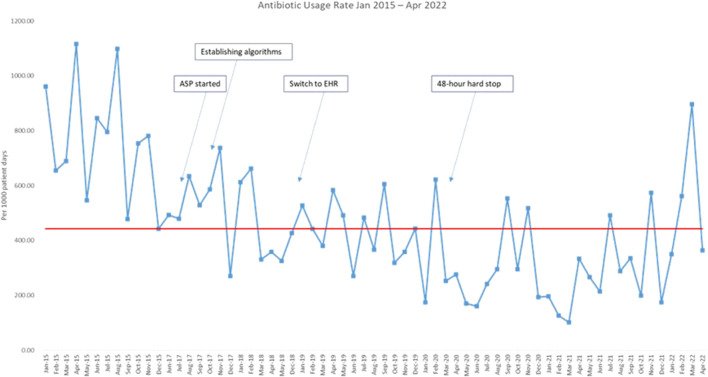
Run chart plotting the antibiotic usage rate from January 2015 till April 2022. Red line: median. Blue line: antibiotic usage rate.ASP: antimicrobial stewardship program; EHR: electronic health record.

The median AUR in the baseline period was 767 per 1,000 patient days (range 441–1,115 per 1,000 patient days). AUR was plotted monthly and reported in the run chart (Figure 2). Upon the start of the project and during PDSA 1, there was an initial reduction of 35% in AUR within the first 3 months. The decreasing trend in antimicrobial usage continued throughout PDSA 2, 3, and 4 and ultimately a 63% reduction in AUR was achieved by the end of the study, with median AUR of 287 per 1,000 patient days in the last 2 years.

**FIGURE 3:**
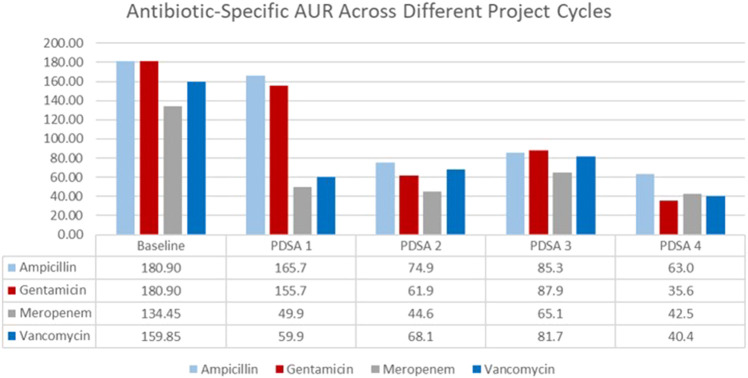
Antibiotic usage rate for the four commonly used antibiotics from January 2015 till April 2022.

AURs for each of the most used antibiotics (ampicillin, gentamicin, vancomycin, and meropenem) are displayed in a bar graph (Figure 3). There was a 64%, 79%, 69%, and 73% reduction in the use of ampicillin, gentamicin, vancomycin, and meropenem, respectively. The highest reduction rate was observed in years three to five of the study, especially after the implementation of the 48-h hard stop.

**FIGURE 4:**
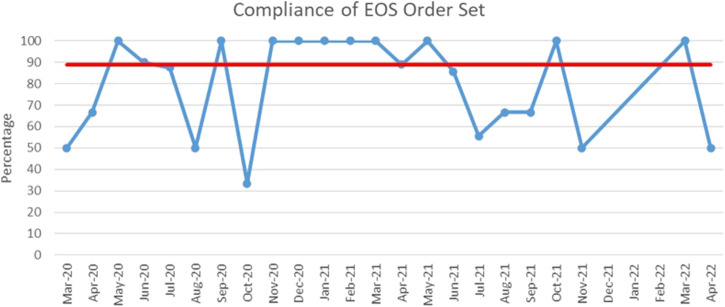
Run chart plotting the usage of the 48-h hard stop order set from March 2020 till April 2022. Red line: median. Blue line: percentage of order set use in case of suspected early onset sepsis.

The process measure of using the 48-h hard stop was tracked with time and median usage was at 88.9% (Figure 4).

Although the mean LOS had increased numerically compared to the baseline (41.6 vs. 23.6 days, *p* = .063), the difference was not statistically significant and was not associated with an increase in antibiotic usage. In fact, this increase was mostly due to a period where the unit was populated by several chronic patients.

## Discussion

This quality improvement project successfully led to the reduction of total as well as antibiotic-specific AURs in the NICU. Within the first few months of the project, and prior to the first practice intervention (PDSA #2), we noted a dip in AUR which is probably due to the Hawthorne effect, a finding that is commonly seen in several quality improvement work.

Baseline AUR at our center is similar to other same-level NICUs in the literature (Araujo da Silva et al., 2020). We show that the implementation of a NS-ASP in a country with no similar programs was achieved successfully and led to culture change, which is evident by maintaining the improvement in the last 2 years of the study even without any new interventions. Our team was able to decrease antibiotic usage by an average of 45% within a duration of 12 months, and to further continue the safe decrease in usage for a total of 5 years. We did not perceive any fading or stagnation of improvement, which could be explained by the smaller number of the end-users, in our case the four neonatologists and three fellows. However, there remains some inter-monthly variation in AUR which could be justified from one clinician to another or the variety and complexity of the clinical cases, whereas the general trend of antibiotic use was persistently decreasing.

We also show in our process measure tracker that there was an acceptable adherence to the use of the 48-h automatic stop order. Usage was at 100% for half of the time of implementation, and more than 50% for most of the months. We also monitored the balancing measures, none of our patients was negatively affected by the intervention and we did not note any inadvertent disruption of planned antibiotic courses. One severely ill patient passed away within hours of birth due to respiratory failure from an MDRO infection, although treatment was not delayed nor affected by the NS-ASP recommendations. Upon further investigation, the maternal placental culture later grew the same organism which was resistant to the routinely recommended combination of ampicillin and gentamicin. We identified three positive blood cultures after implementation of the automatic stop order, all of which were reported positive earlier than the 48 h stop mark: two cultures were positive for group B *streptococcus* after 8 and 10 h, respectively, and one positive for *Escherichia coli* after 6 h.

The project was implemented in a LMIC amidst significant political and economic turmoil which proves that building capacity and sustaining it in the most difficult and challenging circumstances is possible. Lebanon has been passing through a serious economic crisis that has dropped the gross domestic product tremendously and resulted in detrimental socioeconomic effects on healthcare. The high and significant impact of such interventions is well justified by the high prevalence of MDROs in LMIC. Also, applying similar methods could potentially have significant impact in reducing morbidity and mortality secondary to neonatal sepsis. The series of low-cost interventions (excluding human resources), such as formulating treatment algorithms, performing audit and feedback rounds and implementing automatic stop orders for antibiotic prescription, proved to be very effective approaches with high impact on patient outcomes. Our findings suggest that such an endeavor can be generalized to many centers with similar limited resources where newborns with sepsis are being treated. However, the human-resource burden, which might not be available in all settings, might hinder the monitoring and accurate reporting of antibiotic usage. Evidence of successful interventions has been seen in other LMICs, where the implementation of NS-ASP interventions was associated with reduction in antibiotic utilization (Cox et al., 2017; Siachalinga et al., 2022). Although several hurdles are present in such settings, our team was able to mitigate such barriers by efficiently utilizing the EHR to generate automated reports at different intervals, and thus guide the team to decide on the timeliness and appropriateness of the intervention.

Our project carries a lot of strengths. To the best of our knowledge, this is the first NS-ASP implemented in Lebanon and the Middle East with sustainable positive outcomes on AUR reduction. Such a quality improvement approach has been done successfully in different regions of the world reporting similar results to ours. In a prospective quality improvement project, several interventions including standardized approach to care and implementing hard-stops in the NICU resulted in a sustained decreased in AUR of 20%–32% (Agarwal et al., 2021). Another large retrospective cohort study noted significant reductions in AUR in multiple NICUs using several interventions similar to our study (Boverman et al., 2022). The use of an automatic hard stop in the setting of antibiotic stewardship in premature infants has proven to be an optimal strategy to reduce unnecessary antibiotic use (Rajar et al., 2020). We also encountered little resistance to change which was seen by readily adopting new interventions and hints at good buy-in from leadership.

The project had several limitations. The first challenge was the difficulty of getting baseline data as charts at that time were scanned paper documents. This work was time-consuming and might have included recording bias despite the double validation of the manual data. DOT calculations also relied on dispensing pharmacy data and not administration data which could have initially overestimated the usage rates. In the prospective phase of the study, we used medication administration data which was embedded in the EHR to calculate the DOT and thus AUR. We were able to validate both methods for a period of 2 months and got similar results which eliminated the risk for discrepancy between both data sources. All these hindrances were mitigated with the implementation of a new EHR at our institution in November 2018 which created an embedded monthly report to automatically calculate the AUR.

Some challenges, which were beyond our control, were the effect of the Lebanese economic crisis on the healthcare system. During the project, we faced shortages of commonly used antibiotics, mainly ampicillin and flucloxacillin, thus abiding by the recommended algorithms became a demanding task. Another challenge was the COVID-19 pandemic which occurred after 2 years of study implementation. As many other centers, the pandemic affected admission numbers and the acuity of patients admitted to the NICU (Abdul-Mumin et al., 2021; Greenbury et al., 2021; Rasmussen et al., 2021), with both factors influencing DOT and LOS which might have affected the AUR.

In summary, we demonstrated a significant and sustained decrease in antimicrobial use in our study without showing any negative impact on patient clinical outcomes. This promising outcome highlights the need for such approaches when treating newborns in settings similar to ours. The evolving growth of antimicrobial resistance to standard antibiotic regimens in neonates is an increasing problem in LMIC that puts these newborns at increased risk for complications from sepsis, and even death. (Laxminarayan et al., 2016). Reducing and regulating antimicrobial use in NICUs can help halt the development of MDROs and improve outcomes of patients with resistant infections. Another beneficial outcome of reducing antimicrobial use could be seen later in childhood when the detrimental effects of such medications on the developing microbiome is mitigated. Our study shows a promising trend that would lead to beneficial outcomes on the short and long run.

In conclusion, a NS-ASP was successfully implemented and sustained in a LMIC despite facing a multitude of challenges, including the COVID-19 pandemic in addition to economic and political country-specific problems. This model-based and multidisciplinary low-cost approach could lead to marked improvement affecting neonatal outcomes. Our project can be modified and tailored to the needs of other centers facing similar barriers. Further steps will focus on evolving and disseminating this methodology to several centers across the country and the region.

## Data Availability

The raw data supporting the conclusion of this article will be made available by the authors, without undue reservation.
